# Characterization and Phylogenetic Analysis of the Chloroplast Genome of *Carissa spinarum* L. (Rauvolfioideae, Apocynaceae)

**DOI:** 10.1002/ece3.71988

**Published:** 2025-08-14

**Authors:** Yuhua Huang, Hui Li, Abdullah, Sayed Afzal Shah, Irum Naz, Rushan Yan, Bartholomew Yir‐erong, Xiaoxuan Tian

**Affiliations:** ^1^ State Key Laboratory of Chinese Medicine Modernization Tianjin University of Traditional Chinese Medicine Tianjin China; ^2^ Haihe Laboratory of Modern Chinese Medicine Tianjin China; ^3^ Department of Biological Sciences National University of Medical Sciences Rawalpindi Pakistan; ^4^ Department of Biochemistry and Molecular Biology, Institute of Biochemistry, Biotechnology and Bioinformatics, Faculty of Chemical and Biological Sciences The Islamia University of Bahawalpur Bahawalpur Pakistan; ^5^ Institute of Traditional and Alternative Medicine (ITAM), University of Health and Allied Sciences (UHAS) Ho Ghana

**Keywords:** *Carissa*, *Carissa spinarum*, chloroplast genome, phylogeny, Rauvolfioideae, Willughbeieae

## Abstract

*Carissa spinarum* L. (Rauvolfioideae, Apocynaceae), a thorny shrub indigenous to arid regions of South Asia (including Pakistan), is traditionally used to treat fever, diabetes, and inflammation. This study presents the first de novo assembly of the complete chloroplast (cp) genome of *C. spinarum*. The genome comprises 154,654 base pairs (bp) and displays the typical quadripartite structure, consisting of a large single‐copy (LSC) region (84,929 bp), a small single‐copy (SSC) region (18,123 bp), and a pair of inverted repeats (IRa and IRb; 25,801 bp each). Annotation identified 113 unique genes, including 79 protein‐coding genes (CDSs), 30 transfer RNAs (tRNAs), and four ribosomal RNAs (rRNAs), with 16 genes duplicated in the IRs (five CDSs, four rRNAs, and seven tRNAs). Relative synonymous codon usage (RSCU) analysis revealed a strong bias tuoward codons ending in A/T (RSCU ≥ 1), while those ending in C/G were underrepresented (RSCU < 1). Amino acid frequency analysis showed lysine as the most frequently encoded and cysteine as the least abundant. We identified numerous simple sequence repeats (SSRs), with mononucleotide repeats being the most abundant, followed by tetranucleotide repeats, then by trinucleotide repeats; most SSRs were A/T‐rich, consistent with the high overall AT content of the cp genome. Phylogenomic analysis across 19 genera placed *C. spinarum* within the tribe Carisseae, clarified intertribal relationships, and supported the polyphyly of Willughbeieae. As the first cp genome resource for this species, our study provides a valuable foundation for future conservation efforts and evolutionary studies within Rauvolfioideae and the broader Apocynaceae family.

## Graphical abstract

In this study, we present the first de novo assembled chloroplast genome of *Carissa spinarum* L. and provide detailed insights into its genomic features. This is the second genome we are characterizing from the genus Carissa L. as before the article on *Carissa macrocarpa* exists. Furthermore, we reconstructed the phylogenetic relationships of *C. spinarum* with *Carissa macrocarpa* and 18 other species, each representing a different genus within the subfamily Rauvolfioideae (Apocynaceae).

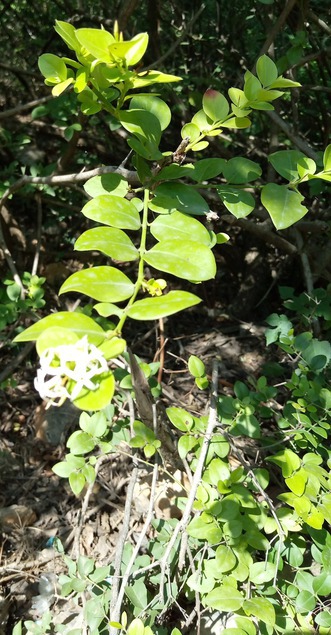

## 1 Introduction

*Carissa* L. (Apocynaceae: Rauvolfioideae), a genus of approximately 15 thorny shrub species, is distributed across tropical Africa and Asia (POWO 2025). *Carissa spinarum* L. (syn. *Carissa carandas* G.Lodd.; locally known as “garna” or “Jungli karonda”) is widely distributed in South Asia, including Pakistan, and typically inhabits arid, rocky regions (POWO 2025; Sharma et al. 2023). This species holds significant ethnopharmacological value; traditional preparations derived from its roots, bark, leaves, stems, and unripe fruits are used to treat diverse ailments, including infections, fever, diabetes, hypertension, arthritis, and wounds (Sharma et al. 2023). The ripe fruits are edible and commonly processed into products such as jams and juices. Phytochemical analyses have identified bioactive compounds—including saponins, alkaloids, tannins, flavonoids, glycosides, and sterols—that underpin its reported anti‐inflammatory, antimicrobial, and antioxidant properties (Sharma et al. 2023).

Advances in high‐throughput sequencing have facilitated comprehensive studies of nuclear, mitochondrial, and chloroplast (cp) genomes, enhancing evolutionary inference and supporting drug discovery (Abdullah et al. 2025; Gao et al. 2023; Li et al. 2024; Zhao et al. 2023). Chloroplast genomes are particularly valuable for plant phylogenetics, evolution, population genetics, conservation, and DNA barcoding due to their uniparental inheritance, lack of recombination, and moderate sequence variation (Abdullah et al. 2021; Ahmed et al. 2020; Abdullah et al. 2025; Li et al. 2024; Teshome et al. 2020). Despite this utility and the species' significance, the complete cp genome of *C. spinarum* remains uncharacterized; only *Carissa macrocarpa* (Eckl.) A.DC. has a published cp genome (Jo et al. 2017). Here, we present the first de novo assembly and characterization of the *C. spinarum* cp genome, providing a key genomic resource for phylogenetic analysis, DNA barcoding, and conservation planning.

## 2 Materials and Methods

### 2.1 Sample Collection and DNA Sequencing

**FIGURE 1:**
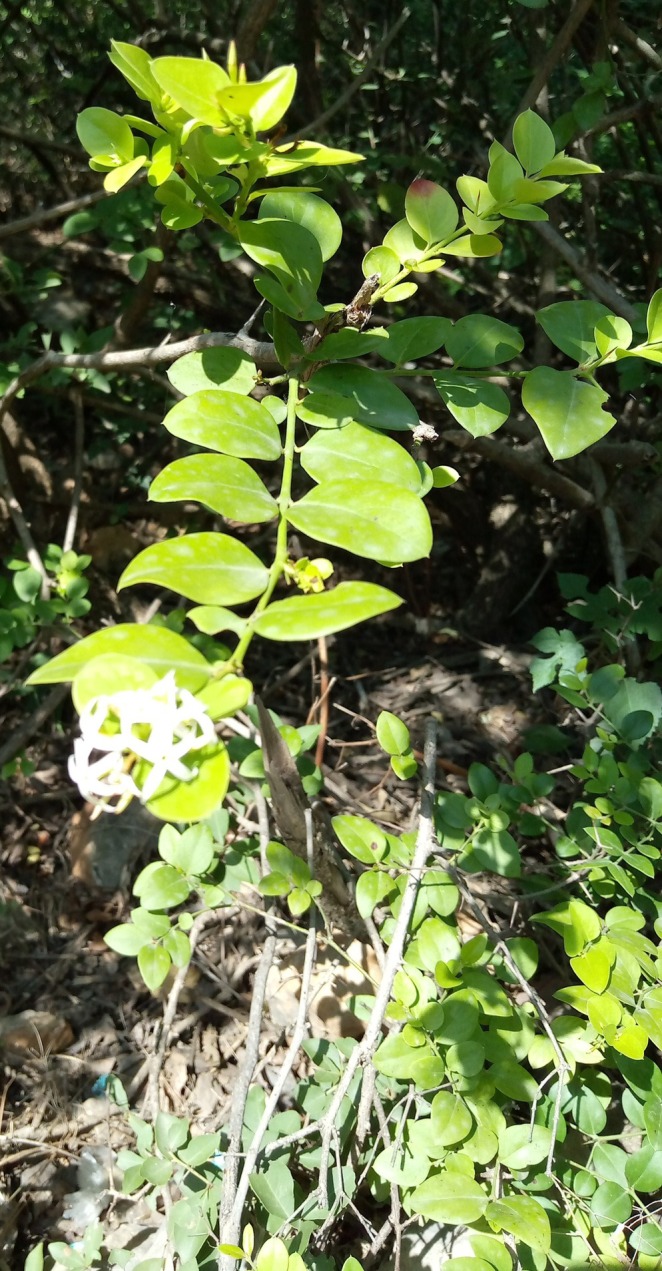
*Carissa spinarum* in its natural habitat. The photograph was taken by Dr. Shah and shows some branches of the plant. Due to the dense vegetation and presence of other plant species in the area, the image focuses on a clearly visible branch of *C. spinarum* for better identification.

*Carissa spinarum* was collected from Quaid‐i‐Azam University, Islamabad, Pakistan (GPS: 33.7478° N, 73.1381° E). The plant was identified by Dr. Shah and deposited in the herbarium of the National University of Medical Sciences, Rawalpindi, under voucher specimen (NUMS00008). A photograph of the plant is provided in Figure 1. No permission was required from local authorities for the collection and use of this plant in genomic research. Genomic DNA was extracted from silica‐dried leaves using the Plant Genomic DNA Kit (Tiangen Biotech) following the manufacturer's protocol.

After extraction, DNA quantity, integrity, and purity were assessed by Novogene (Tianjin, China) using an Agilent 5400 system. After quality confirmation, high‐quality DNA was randomly fragmented using a Covaris ultrasonicator. Library preparation included end repair, A‐tailing, adapter ligation, fragment size selection, PCR amplification, and purification. Insert size was verified using an AATI Fragment Analyzer, and effective library concentration was measured by qPCR. Finally, the library that passed quality thresholds was sequenced using 150 bp paired‐end reads on the Illumina NovaSeq 6000 platform.

Quality filtering of raw reads was performed using fastp v0.23.1 (Chen et al. 2018) with the following parameters: ‐g ‐q 5 ‐u 50 ‐n 15 ‐l 150 ‐‐overlap_diff_limit 1 ‐‐overlap_diff_percent_limit 10. Paired reads were discarded if either read contained more than 10% ambiguous bases (N), more than 50% low‐quality bases (*Q* ≤ 5), or adapter contamination. Only high‐quality clean reads were retained for downstream analyses, with PHRED quality scores of *Q*20 and *Q*30 reaching 98.86% and 96.9%, respectively.

### 2.2 De Novo Assembly of the Chloroplast Genome of *Carissa spinarum* and Annotations

GetOrganelle v1.7.7.0 (Jin et al. 2020) was used for de novo assembly of the cp genome from clean reads under default parameters. Both GeSeq v2.0.3 (Tillich et al. 2017) and PGA v1 (Qu et al. 2019) were used for annotations, while tRNAscan‐SE v2.0.7 (Chan and Lowe 2019) and ARAGORN v1.2.38 (Laslett and Canback 2004) were employed to further verify tRNA genes. Coding sequences were manually curated in Geneious R8.1 by aligning with reference cp genomes ( *C. macrocarpa* : KX364402; *Cerbera manghas* L.: NC_051546) using MAFFT v7 (Katoh and Standley 2013) and correcting start and stop codons.

### 2.3 Chloroplast Genome Features and Phylogenetic Analysis

Assembly accuracy was assessed by mapping raw reads to the assembled genome in Geneious R8.1 (Kearse et al. 2012), with coverage depth calculated. Genome structure features (IRs, SSC, LSC) were analyzed in Geneious, and a circular map was generated using Chloroplot (Zheng et al. 2020). The gene content and gene arrangement were also compared with the previously reported cp genome of *C. macrocarpa* through Mauve alignment (Darling et al. 2004). Figures illustrating cis‐spliced genes and trans‐spliced genes were generated using CPGview (Liu et al. 2023). Relative synonymous codon usage (RSCU) and amino acid frequencies were calculated using custom Python scripts (Script 1 and Script 2 in Data S1).

Simple sequence repeats (SSRs) were identified using the MISA‐web tool (Beier et al. 2017). The minimum thresholds for repeat motifs were set as follows: 10 for mononucleotide, 5 for dinucleotide, 4 for trinucleotide, and 3 for tetra‐, penta‐, and hexanucleotide repeats.

Complete cp genomes of 20 Rauvolfioideae (Apocynaceae) species and one outgroup (*Mitrasacme pygmaea* R.Br.: NC050922) were retrieved from NCBI. Following IRa region removal, sequences were aligned with MAFFT. Fast‐evolving and ambiguously aligned regions were manually excluded, resulting in a final dataset comprising 21 taxa and 76,735 sites. Phylogenetic reconstruction was performed in IQ‐TREE v3 (Hoang et al. 2018; Minh et al. 2020) with 10,000 ultrafast bootstrap replicates and SH‐aLRT tests under the optimal model (GTR + F + I + R3) as selected via ModelFinder (Kalyaanamoorthy et al. 2017). The Interactive tree of Life was used online to draw phylogeny and improve tree visualization (Letunic and Bork 2019).

## 3 Results and Discussion

### 3.1 Features of *Carissa Spinarum* Chloroplast Genome

Whole‐genome sequencing of *C. spinarum* yielded 55.25 million clean paired‐end reads (~19.2 GB raw data). The high depth (average coverage: 6134×) enabled robust de novo assembly of the complete cp genome, with high accuracy and completeness confirmed through read mapping validation.

**FIGURE 2:**
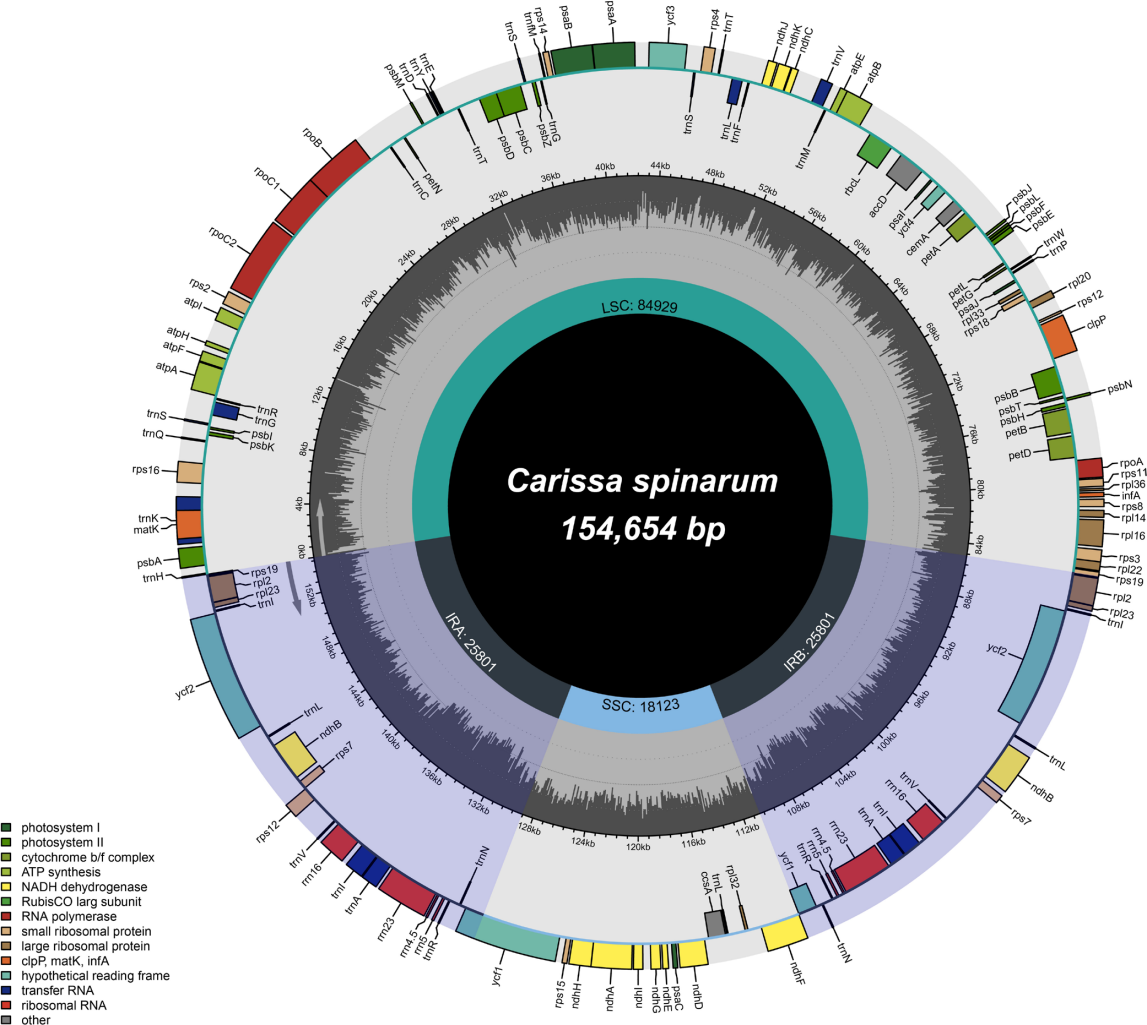
Circular map of the chloroplast genome of *Carissa spinarum* (154,654 bp). The genome exhibits a typical quadripartite structure, comprising a large single‐copy (LSC) region (84,929 bp), a small single‐copy (SSC) region (18,123 bp), and two inverted repeat (IRa and IRb) regions (25,801 bp each). Genes are color‐coded by functional categories as indicated in the legend. Genes transcribed in the clockwise direction are shown on the inside of the outer circle, while those transcribed counterclockwise are depicted on the outside. The inner circle represents the GC content variation across the genome, with the darker areas indicating higher GC content. Arrows on gene blocks indicate the direction of transcription.

**FIGURE 3:**
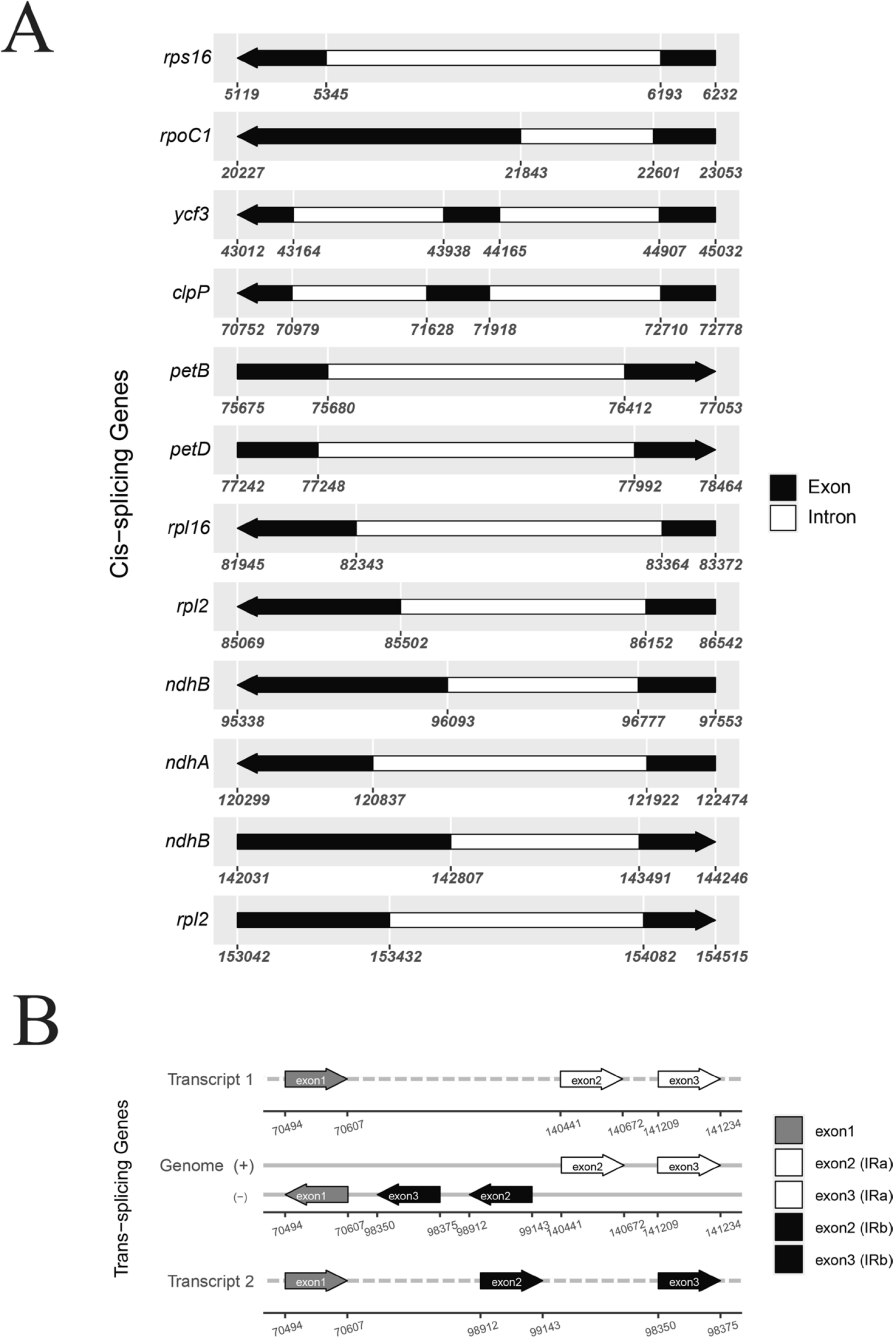
Gene structure map of *cis*‐ and *trans*‐spliced genes in the *C. spinarum* chloroplast genome. (A) *Cis*‐spliced genes. (B) *Trans*‐spliced genes.

The assembled *C. spinarum* cp genome was 154,654 bp long and exhibited the characteristic quadripartite structure of angiosperms (Figure 2), comprising a large single‐copy (LSC) region (84,929 bp), a small single‐copy (SSC) region (18,123 bp), and two inverted repeat regions (IRa/IRb; 25,801 bp each). The previously reported cp genome of *C. macrocarpa* was 155,297 bp long and exhibited the same structural organization, comprising an LSC region (85,582 bp), an SSC region (18,129 bp), and IRa and IRb each 25,793 bp. In both species, the cp genome contained 113 unique genes: 79 protein‐coding (CDS), 30 transfer RNA (tRNA) genes, and four ribosomal RNA (rRNA) genes (Table 1). In the IR regions, sixteen duplicated genes were found, including five CDS genes (*ndhB*, *rpl2*, *rpl23*, *rps7* and *ycf2*), four rRNA genes (*rrn16S*, *rrn23S*, *rrn4.5S*, *rrn5S*), and seven tRNA genes (*trnA‐UGC*, *trnI‐GAU*, *trnL‐CAA*, *trnN‐GUU*, *trnR‐ACG*, *trnV‐GAC*, *trnI‐CAU*). Eighteen genes contained introns: 12 CDS (*atpF*, *petB*, *rps16*, *rps12*, *rpl2*, *ndhA*, *rpl16*, *ndhB*, *rpoC1*, *ycf3*, *petD*, *clpP*) and six tRNAs (*trnV‐UAC*, *trnK‐UUU*, *trnG‐UCC*, *trnI‐GAU*, *trnL‐UAA*, *trnA‐UGC*). Among the CDS genes, two genes (*ycf3* and *clpP*) contained two introns each, while ten genes had one intron (Figure 3A). The *rps12* gene was trans‐spliced with exon 1 in the LSC region and exons 2 and 3 in the IR regions (Figure 3B). The analysis based on Mauve progressive alignment revealed not only similar gene content but also gene arrangement between the two species (Figure S1). Our results are in agreement with previous studies of Apocynaceae in terms of unique gene content (Odago et al. 2022; Rodda and Niissalo 2021; Straub et al. 2011; Wang et al. 2023; Zhang et al. 2023). However, some genera show a high level of IR expansion that leads to an increase in gene content to 142, as reported in the *Hoya* group (Odago et al. 2022; Rodda and Niissalo 2021), whereas the number of genes in our study is consistent with those genera of Apocynaceae that showed conserved IR contraction and expansion and did not exhibit IR expansion that increases gene number (Wang et al. 2023).

**TABLE 1:** Functional classification of genes in the chloroplast genome of *Carissa spinarum*.

Category for genes	Group of genes	Name of genes	Amount
Self‐replication	Large subunit of ribosome (LSU)	*rpl14*, rpl16*, rpl2* ^a^, *rpl20*, *rpl22*, *rpl23* ^a^, *rpl32*, *rpl33*, *rpl36*	11
	Small subunit of ribosome (SSU)	*rps11*, rps12*, *rps14*, *rps15*, rps16*, *rps18*, *rps19*, *rps2*, *rps3*, *rps4*, *rps7* ^a^, *rps8*	13
	DNA dependent RNA polymerase	*rpoA*, *rpoB*, rpoC1*, *rpoC2*	4
	rRNA genes	*rrn16* ^a^, *rrn23* ^a^, *rrn4.5* ^a^, *rrn5* ^a^	8
	tRNA genes	*trnA‐UGC* ^a^, *trnC‐GCA*, *trnD‐GUC*, *trnE‐UUC*, *trnF‐GAA*, *trnG‐GCC*, *trnG‐UCC*, *trnH‐GUG*, *trnI‐CAU* ^a^, *trnI‐GAU* ^a^, *trnK‐UUU*, *trnL‐CAA* ^a^, *trnL‐UAA*, *trnL‐UAG*, *trnM‐CAU*, *trnN‐GUU* ^a^, *trnP‐UGG*, *trnQ‐UUG*, *trnR‐ACG* ^a^, *trnR‐UCU*, *trnS‐GCU*, *trnS‐GGA*, *trnS‐UGA*, *trnT‐GGU*, *trnT‐UGU*, *trnV‐GAC* ^a^, *trnV‐UAC*, *trnW‐CCA*, *trnY‐GUA*, *trnfM‐CAU*	37
Photosynthesis	Photosystem I	*psaA*, *psaB*, *psaC*, *psaI*, *psaJ*	5
	Photosystem II	*psbA*, *psbB*, *psbC*, *psbD*, *psbE*, *psbF*, *psbH*, *psbI*, *psbJ*, *psbK*, *psbL*, *psbM*, *psbN* (*pbf1*), *psbT*, *psbZ*	15
	NADPH dehydrogenase	ndhA*, ndhB* ^a^, *ndhC*, *ndhD*, *ndhE*, *ndhF*, *ndhG*, *ndhH*, *ndhI*, *ndhJ*, *ndhK*	12
	Cytochrome b/f complex	*petA*, petB*, petD*, *petG*, *petL*, *petN*	6
	Subunits of ATP synthase	*atpA*, *atpB*, *atpE*, *atpF*, *atpH*, *atpI*	6
	Large subunit of Rubisco	*rbcL*	1
	Photosystem I assembly proteins	ycf3* (*pafI*), *ycf4* (*pafII*)	2
Other genes	Protease	clpP*	1
	Maturase	*matK*	1
	Envelop membrane protein	*cemA*	1
	Subunit of acetyl‐CoA‐carboxylase	*accD*	1
	C‐type cytochrome synthesis gene	*ccsA*	1
	Translation initiation factor	*infA*	1
	Conserved open reading frames	*ycf1*, *ycf2* ^a^	3
		Total number of genes	129

*Note:* *gene containing introns, ^a^gene duplicating in inverted repeats; *pafI*, *pafII*, and *psbf1* were listed in parentheses, as GeSeq annotated the genes *ycf3*, *ycf4*, and *psbN* under these names, respectively.

The GC content analysis revealed high similarities between both species. The overall GC content was 38.1%, with regional variation: LSC (36.2%), IRs (43.3%), and SSC (32.1%). Among genes, the GC content of rRNA was highest (55.4%), followed by tRNAs (53.4%) and then CDS (38.4%). This distribution aligns with patterns observed in Apocynaceae and other plant lineages (Abdullah et al. 2021; Henriquez et al. 2020; Wang et al. 2023; Zhang et al. 2024).

### 3.2 Codon Usage, Amino Acid Frequency, and Simple Sequence Repeats Analysis

**FIGURE 4:**
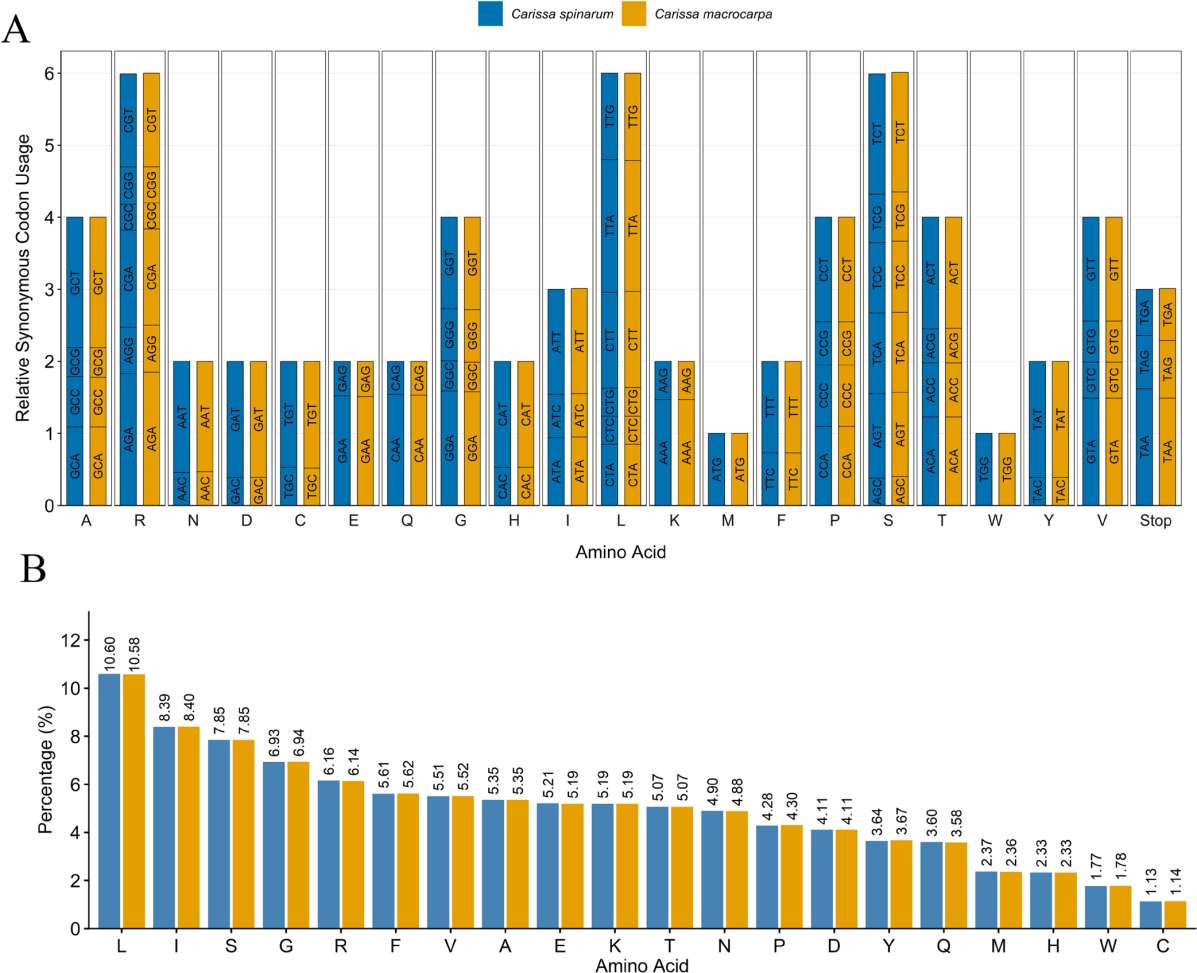
Codon usage and amino acid frequency in the chloroplast genomes of *C. spinarum* and *C. macrocarpa* . (A) Relative synonymous codon usage (RSCU). Amino acids are indicated on the *x*‐axis, and the height of each bar represents the RSCU value for each species. Codons are labeled within the bars. (B) Amino acid frequency distribution, showing the proportion of each amino acid encoded in the chloroplast genomes.

We conducted a comparative analysis of gene arrangement, codon usage, amino acid frequency, and simple sequence repeats (SSRs) between the two *Carissa* species and observed a high degree of similarity across all parameters. Codon usage patterns revealed a strong bias toward codons ending in adenine (A) or thymine (T) at the third position, as indicated by relative synonymous codon usage (RSCU) values greater than 1. In contrast, codons ending in cytosine (C) or guanine (G) typically had RSCU values less than 1 (Figure 4A). Among the 20 amino acids, leucine exhibited the highest frequency in codon representation, while cysteine showed the lowest (Figure 4B). Notably, leucine was encoded by six synonymous codons, reflecting a pronounced codon usage bias and contributing to its elevated frequency. These results are in agreement with previous studies of Apocynaceae and other plant lineages (Abdullah et al. 2021; Li et al. 2024; Zhang et al. 2024).

**FIGURE 5:**
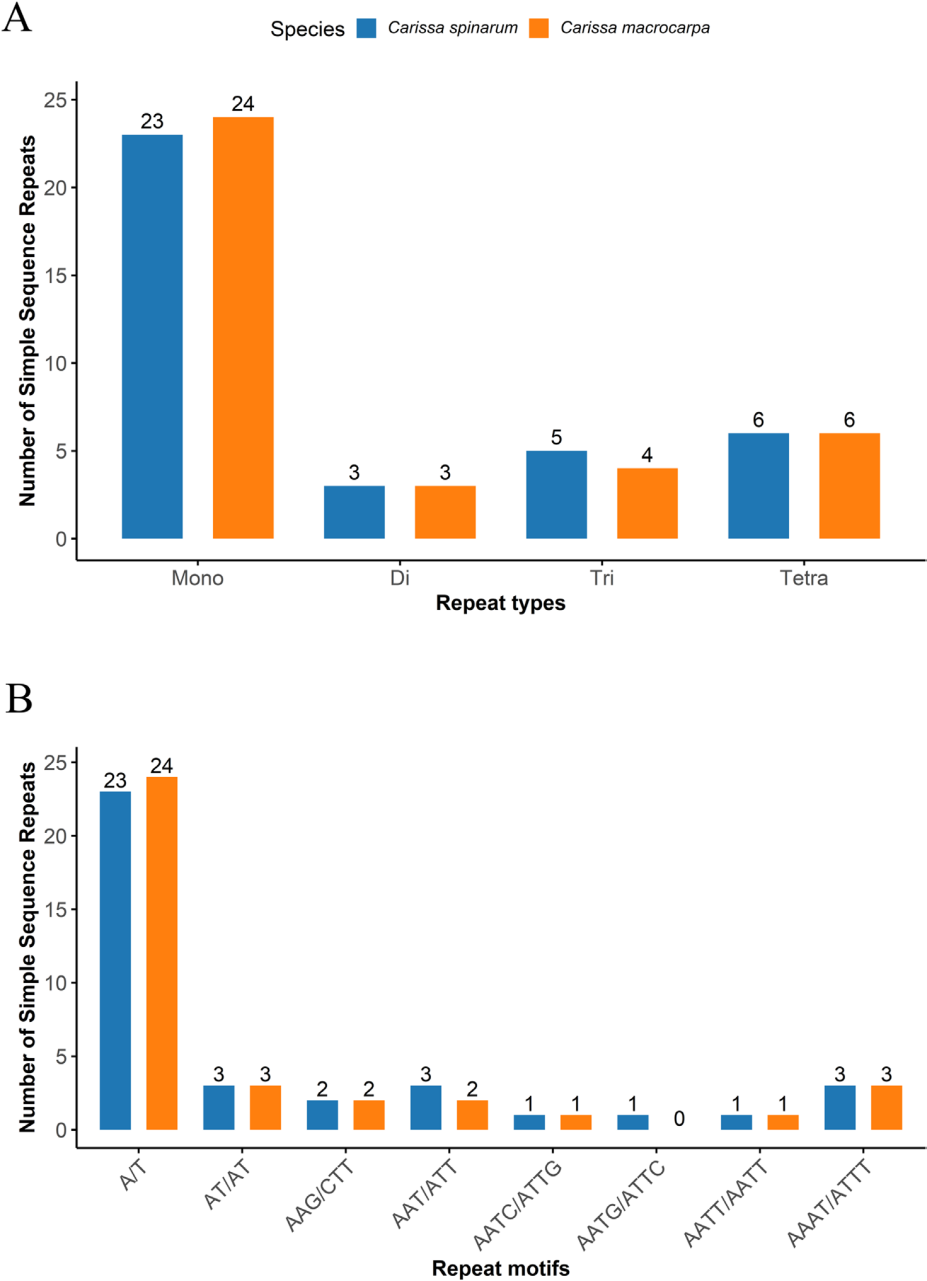
Simple sequence repeat (SSR) analysis of the *C. spinarum* and *C. macrocarpa* chloroplast genome. (A) Identified SSR types. (B) SSR motif compositions.

SSR analysis revealed that mononucleotide repeats accounted for the largest proportion of SSRs, followed by tetranucleotide and trinucleotide repeats, with both species displaying a consistent distribution pattern (Figure 5A). Most SSRs were composed of A/T‐rich motifs (Figure 5B), reflecting the characteristically high AT content of chloroplast genomes (Abdullah et al. 2021; Li et al. 2024; Zhang et al. 2024). Given their abundance and variability, these SSRs are potentially valuable for population genetic studies, as chloroplast SSRs have previously demonstrated high efficacy in such analyses (Huang et al. 2011).

### 3.3 Phylogenetic Placement of *Carissa spinarum* and Analysis of Related Genera and Tribes

**FIGURE 6:**
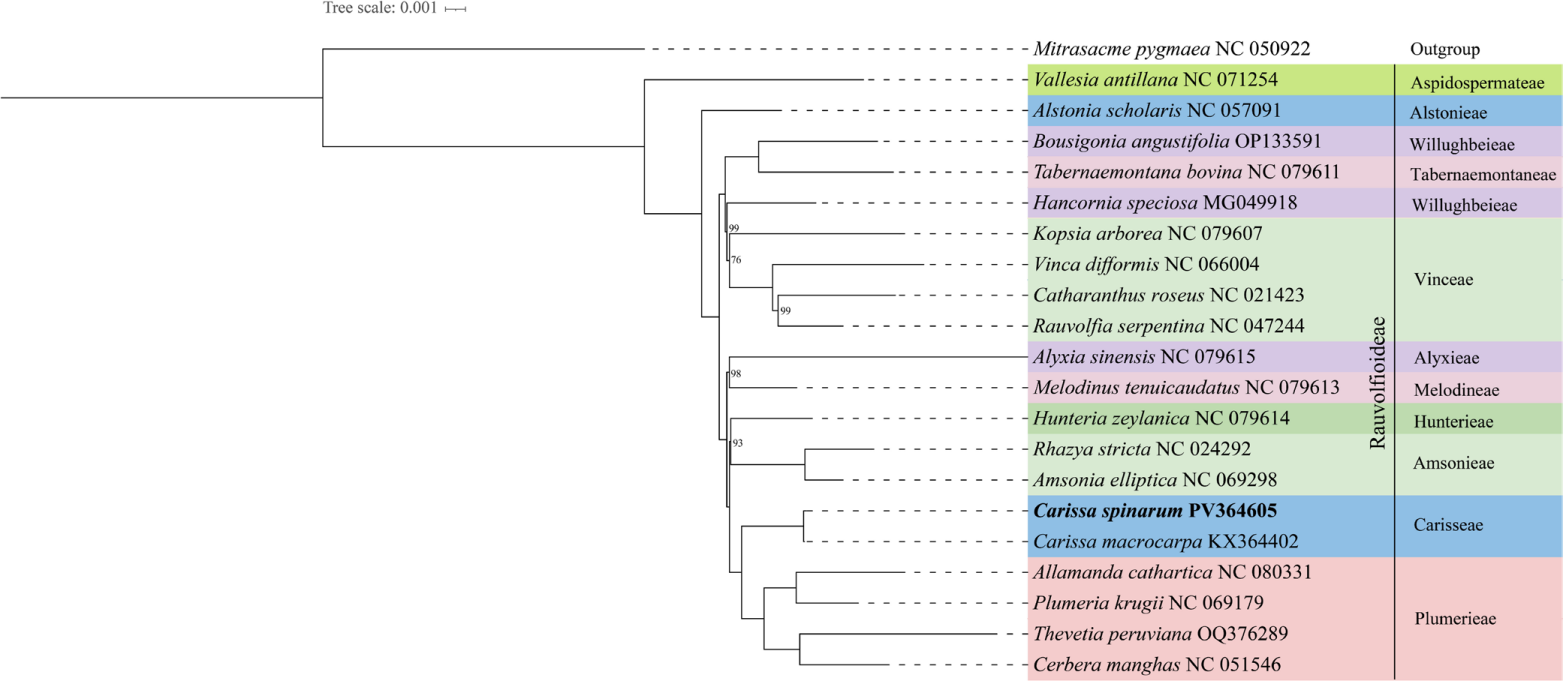
Maximum likelihood phylogenetic tree of 21 Rauvolfioideae species. The newly sequenced *C. spinarum* is highlighted in bold. *Mitrasacme pygmaea* served as the outgroup. Bootstrap support values (< 100%) are indicated at nodes; values of 100% are omitted for clarity.

Phylogenetic reconstruction based on complete cp genome sequences produced a highly resolved topology with strong bootstrap support across most nodes (Figure 6), providing robust insights into intertribal relationships within Rauvolfioideae (Apocynaceae). The tree was rooted with *Mitrasacme pygmaea* (NC050922), serving as an outgroup.

Among the sampled lineages, *Vallesia antillana* Woodson (tribe Aspidospermateae) occupied a basal position, diverging earliest from the remaining taxa. The next divergence involved *Alstonia scholaris* (L.) R.Br. (Alstonieae), forming a sister lineage to a well‐supported clade comprising the remaining tribes.

Within this major clade, Plumerieae ( *Plumeria krugii* Urb., *Cascabela thevetia* (L.) Lippold, *Allamanda cathartica* L.) and Carisseae ( *C. macrocarpa* , *C. spinarum*) formed strongly supported sister groups, confirming the close relationship between *Carissa* species and other members of Plumerieae. Similarly, *Amsonia elliptica* (Thunb.) Roem. and Schult. (Amsonieae) and *Hunteria zeylanica* (Retz.) Gardner ex Thwaites (Hunterieae) were grouped as sister taxa, representing early‐diverging lineages. *Melodinus tenuicaudatus* Tsiang and P.T.Li (Melodineae) and *Alyxia sinensis* Champ. ex Benth. (Alyxieae) also formed a well‐supported sister group.

Notably, the tribe Vinceae—comprising *Catharanthus roseus* (L.) G.Don, *Vinca difformis Pourr*., and *Rauvolfia serpentina* (L.) Benth. ex Kurz—formed a cohesive clade that was sister to *Hancornia speciosa* Gomes (Willughbeieae). Interestingly, Willughbeieae also formed a sister relationship with *Tabernaemontana bovina* Lour. (Tabernaemontaneae), suggesting a potential polyphyletic origin of Willughbeieae within this plastome‐based phylogeny.

Overall, the recovered topology supports most current tribal circumscriptions, including Plumerieae, Carisseae, Amsonieae, Hunterieae, Melodineae, Alyxieae, Vinceae, Tabernaemontaneae, Alstonieae, and Aspidospermateae. These findings are consistent with previous plastome‐based phylogenomic analyses (Wang et al. 2023), which also reported polyphyletic relationships within Willughbeieae. Our results corroborate these observations and further underscore the need to reassess the tribal limits of Willughbeieae using broader taxon sampling and integrating nuclear and cp genome data.

## 4 Conclusion

We de novo assembled and characterized the cp genome of *C. spinarum*. Phylogenomic analysis confirmed its placement within the tribe Carisseae and provided a well‐resolved framework of intertribal relationships in Rauvolfioideae, including evidence supporting the potential polyphyly of Willughbeieae. This newly sequenced chloroplast genome may serve as a valuable resource for future taxonomic, phylogenetic, and evolutionary studies within the subfamily Rauvolfioideae and the broader Apocynaceae family.

## Author Contributions

**Yuhua Huang:** conceptualization (equal), data curation (equal), formal analysis (equal), writing – original draft (equal). **Hui Li:** conceptualization (equal), data curation (equal), formal analysis (equal), writing – original draft (equal). data curation (equal), formal analysis (equal), methodology (equal), visualization (equal), writing – original draft (equal). **Sayed Afzal Shah:** data curation (equal), resources (equal). **Irum Naz:** data curation (supporting), investigation (equal). **Rushan Yan:** data curation (equal), writing – review and editing (equal). **Bartholomew Yir‐erong:** conceptualization (equal), investigation (equal), methodology (equal), resources (equal), writing – review and editing (equal). **Xiaoxuan Tian:** investigation (equal), methodology (equal), resources (equal), supervision (equal), writing – review and editing (equal).

## Ethics Statement

This study was conducted in full compliance with local and international regulations governing plant research. No specific permits were required for the collection of plant material or for conducting genomic research on the species studied.

## Consent

All authors agreed upon the content of the manuscript.

## Conflicts of Interest

The authors declare no conflicts of interest.

## Supporting information


**Figure S1:** The Mauve progressive alignment revealed high conservation of cp genome between *C. spinarum* and *C. macrocarpa* .

Data S1:

## Data Availability

The assembled chloroplast genome sequence from this study has been deposited in NCBI under the accession number PV364605; the associated raw sequencing data are available under BioProject PRJNA1275558.
