# Supplementary figures and images for: Characterization and Phylogenetic Analysis of the Chloroplast Genome of Carissa spinarum L. (Rauvolfioideae, Apocynaceae)
Source: Ecol Evol. 2025 Aug 14;15(8):e71988. doi: 10.1002/ece3.71988 (PMC12354977; doi:10.1002/ece3.71988)

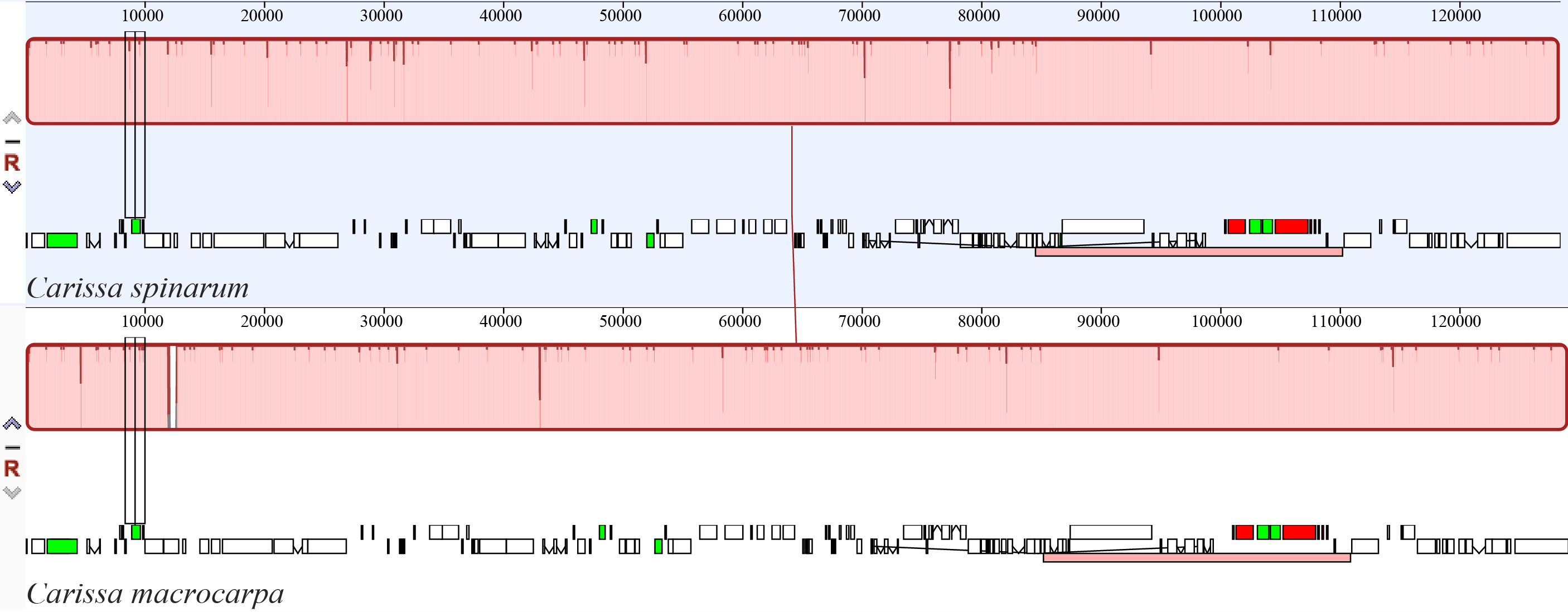

Supplement: Supplementary file 1 — Figure S1: The Mauve progressive alignment revealed high conservation of cp genome between C. spinarum and C. macrocarpa . [file ECE3-15-e71988-s001.jpg]
